# Sex Differences in Adipose Tissue CD8^+^ T Cells and Regulatory T Cells in Middle-Aged Mice

**DOI:** 10.3389/fimmu.2018.00659

**Published:** 2018-04-04

**Authors:** Hilda Ahnstedt, Meaghan Roy-O’Reilly, Monica S. Spychala, Alexis S. Mobley, Javiera Bravo-Alegria, Anjali Chauhan, Jaroslaw Aronowski, Sean P. Marrelli, Louise D. McCullough

**Affiliations:** Department of Neurology, McGovern Medical School at University of Texas Health Science Center at Houston (UTHealth), Houston, TX, United States

**Keywords:** sex differences, aging, inflammation, CD8^+^ T cells, adipose tissue, regulatory T cells

## Abstract

The prevalence of cardiovascular disease has increased among middle-aged women in the United States, yet has declined in middle-aged men. In experimental stroke, middle-aged females have larger strokes and greater inflammation than age-matched males or younger females. The mechanism underlying this shift from an “ischemia-protected” to an “ischemia-sensitive” phenotype in aging females is unknown. One potential factor is an age-related increase in systemic factors that induce inflammation. Increased abdominal fat deposition is seen in women during middle age. Adipose tissue plays a key role in obesity-induced systemic inflammation, including increased pro-inflammatory cytokines. We hypothesized that age and sex differences in adipose immune cells promote an augmented pro-inflammatory milieu in middle-aged females driven by a balance shift between pro-inflammatory and anti-inflammatory T cells. Abdominal adipose tissue immune cells from young (3–4 months) and middle-aged (15–16 months) male and female C57BL/6J mice were analyzed by flow cytometry. Plasma triglyceride (TG), high-density lipoprotein (HDL), and low-density lipoprotein (LDL) levels were determined with colorimetric assays. Middle-aged mice had higher adipose tissue mass compared to young mice. Lipid profiling showed no sex differences in TG and LDL, but middle-aged females had lower HDL (0.84 ± 0.07 μg/μl) than middle-aged males (1.35 ± 0.06 μg/μl). Flow cytometry data demonstrated an age-associated increase in adipose tissue CD8^+^ T cells that was augmented by female sex, with middle-aged females having a higher percentage of CD8^+^ cells (34.4 ± 3.2% of CD3^+^ T cells) than middle-aged males (24.4 ± 2.2%). This increase in CD8^+^ T-cell proportion was adipose tissue-specific, as this change was not observed in blood. Middle-aged females had higher numbers of activated (CD69^+^) CD8^+^ T cells than males. In addition, female CD8^+^ T cells produced higher levels of IFN-γ, TNF-α, and granzyme B *ex vivo*, and females had higher adipose levels of IFN-γ, RANTES and MIP-1β than middle-aged males. In parallel, females had lower levels of regulatory T cells (Tregs), an anti-inflammatory T-cell subtype, compared to age-matched males. In conclusion, middle-aged females have a detrimental combination of elevated pro-inflammatory T cells and decreased anti-inflammatory Tregs in adipose tissue, which may promote a pro-inflammatory milieu and contribute to increased cardiovascular disease burden in aging females.

## Introduction

Aging represents the largest risk factor for cardiovascular disease. Women experience increased cardiovascular disease and elevated stroke risk in middle-age, while the prevalence in similarly aged men decreases (1, 2). Our previous studies have shown that while young female mice have smaller infarcts after an experimental stroke compared to males, this phenotype is reversed in middle-aged animals (3). The underlying mechanism behind this change from an “ischemia-protected” to an “ischemia-sensitive” phenotype in middle-aged females is unknown. One potential factor may be the age-related increase in adipose tissue in women during menopause, leading to increased adipose tissue inflammation and an enhanced systemic pro-inflammatory environment prior to the stroke. Obesity is a major health problem and a well-known predictor of cardiovascular disease in both sexes. While some cardiovascular risk factors, such as heart disease, are more prevalent in men, abdominal obesity is 2–10 times more common in women in many parts of the world (4–6). In particular, the menopausal transition is associated with a significant increase in body weight and abdominal fat in middle-aged females (7). In addition, studies suggest that abdominal obesity may contribute to a greater risk for ischemic stroke in women than in men (1, 8, 9). The reasons for the sex differences in obesity and risk of stroke in middle-aged men and women are not fully understood.

Adipose tissue is now recognized as an endocrine organ that plays a key role in obesity-induced systemic inflammation. Obesity-induced inflammation is characterized by the infiltration and retention of immune cells within the adipose tissue and the chronic release of pro-inflammatory cytokines, including TNF-α, IL-1β, IFN-γ, and IL-6 (10, 11). Infiltrating immune cells release cytokines, chemokines, metalloproteinases, and reactive oxygen species. This obesity-induced, low-grade systemic inflammation has been linked to insulin resistance, diabetes, arterial stiffness, endothelial dysfunction, and increased blood–brain barrier permeability (12–14). Aging by itself is characterized by a state of chronic inflammation, known as “inflammaging,” and obesity superimposed on aging represents an additional risk factor for chronic disease and age-related complications.

Pro-inflammatory CD8^+^ T cells and anti-inflammatory regulatory T cells (Tregs) are immune cells that normally are found in adipose tissue. The balance between these cells is believed to be an important contributor to obesity. The number of adipose CD8^+^ effector T cells is increased in obesity and CD8^+^ T cells have further been shown to initiate and propagate adipose inflammation by the recruitment and activation of macrophages (15, 16). Conversely, levels of anti-inflammatory Tregs are decreased in genetic- and diet-induced mouse models of obesity (17). Importantly, studies in human subjects also suggest that obesity influences adipose T-cell subset composition and activation. Compared to normal weight patients, obese human subjects have lower levels of circulating Tregs (18). In addition, increased waist circumference has been shown to influence the activation status of both CD4^+^ and CD8^+^ T cells (19). Furthermore, studies have shown that women may be at a greater risk for secondary health issues arising from obesity (1, 8, 9, 20).

The vast majority of experimental studies on adipose tissue inflammation and T-cell immune responses have been performed using genetic- or diet-induced obesity in young male animals. However, as age is a major driver of both obesity and risk for cardiovascular disease in humans, understanding the effects of aging on immune responses within the adipose tissue is important. Because wild-type mice tend to develop natural increases in adiposity and body weight as they age, this study utilized middle-aged mice as a translational natural model of obesity-induced adipose tissue inflammation. Here, we used young and middle-aged mice of both sexes to characterize the intersectional effects of sex and aging on adipose tissue mass, immune cell composition, pro-inflammatory responses, and lipid profile.

## Materials and Methods

### Animals

Young (3–4 months) and middle-aged (15–16 months, aged in-house) C57BL/6J mice were purchased from The Jackson Laboratory (000664; Bar Harbor, ME, USA). All animals had access to chow and water *ad libitum*. Animal procedures were performed in accordance with National Institutes of Health Guidelines for the care and use of laboratory animals and approved by the Animal Welfare Committee at the University of Texas Health Science Center at Houston, TX, USA (AWC-15-0140).

### Estrus Cycle Characterization in Young and Middle-Aged Female Mice

The estrus cycle in young and middle-aged female mice was monitored daily through collection of vaginal smears and examination of the types of cells present for two to three consecutive cycles as described previously (21, 22).

### Tissue Harvesting

Mice were euthanized by Avertin injection and blood was collected by cardiac puncture using heparin-coated needles. For plasma collection, blood was centrifuged (10,000 × *g* for 10 min at 4°C) and the plasma supernatant was removed and stored at −80°C until use. Mice were then transcardially perfused with 60 ml cold, sterile PBS and perigonadal white adipose tissue (epididymal in males and parametrial in females, 300 mg) was carefully dissected for use in downstream applications. Uteri were collected in young and middle-aged female mice and the wet-weights recorded.

### High-Density Lipoprotein (HDL), Low-Density Lipoprotein (LDL), and Triglyceride (TG) Assays

High-density lipoprotein and LDL concentrations in plasma were determined using colorimetric assays from Abcam (Cambridge, MA, USA) according to the manufacturer’s instructions. Briefly, plasma was diluted 1:1 in precipitation buffer and incubated for 10 min at room temperature, followed by centrifugation (2,000 × *g* for 10 min). The supernatant containing the HDL fraction and the LDL precipitate were then incubated for 60 min at room temperature with cholesterol reaction mixture and read on a microplate reader at OD 570 nm. For TG measurements, plasma was diluted 1:5 in TG assay buffer and TG was converted to glycerol and fatty acid by the addition of lipase. Following incubation with TG reaction mixture for 60 min at room temperature, plates were read at OD 570 nm.

### Flow Cytometry

Adipose tissue was mechanically disrupted followed by digestion with collagenase II (C6885, 1 mg/ml, Sigma-Aldrich, St. Louis, MO, USA) at 37°C and 200 rpm for 45 min. EDTA (10 mM) was added during the last 5 min to facilitate dissociation of leukocytes from the adipocytes. The cell suspension was filtered through a 70-µm filter and Fc receptors were blocked with CD16/32 (BioLegend, San Diego, CA, USA) prior to staining of surface markers. Cells were stained for viability (Fixable Live/Dead Aqua Stain, Thermo Fisher Scientific, Waltham, MA, USA) for 30 min, followed by incubation with primary antibodies (CD45-BV605, CD8-BV421, and CD69-PE-Cy7 from Biolegend; and CD11b-PerCP-Cy5.5, CD4-APC, CD25-FITC, and CD3-APC-Cy7 from TONBO Biosciences, San Diego, CA, USA) for 30 min at room temperature. Subsequently, leukocytes were fixed and permeabilized with FoxP3 staining buffer set (eBioscience, Thermo Fisher Scientific) and stained with FoxP3-PE (eBioscience, Thermo Fisher Scientific) for 45 min at room temperature. Leukocytes were re-suspended in FACS buffer and count bright counting beads (Thermo Fischer Scientific) were added prior to reading in a Cytoflex S flow cytometer (Beckman-Coulter, Brea, CA, USA).

For intracellular cytokine staining, leukocytes were isolated as described above and 1 × 10^6^ cells were incubated in complete RPMI-1640 containing Brefeldin A (Golgiplug, Thermo Fisher Scientific). Cells were then stimulated with Cell Stimulation Cocktail (eBioscience, Thermo Fisher Scientific) containing phorbol 12-myristate 13-acetate (PMA, 50 ng/ml) and ionomycin (0.95 µg/ml) or PBS (no stimulation control) and incubated for 4 h at 37°C (5% CO_2_). Following Fc block and surface antigen staining (CD8-BV605, CD45-FITC, CD3 PerCP-Cy5.5, CD4-PE-Cy7, and CD11b-APC-Cy7), cells were fixed and permeabilized (BD Biosciences, San Jose, CA, USA). Cells were then stained for intracellular cytokines (IFN-γ-BV421, TNF-α-APC, granzyme B (GzmB)-PE, Biolegend) for 30 min on ice prior to flow cytometric analysis.

### Multiplex Cytokine Measurement

Adipose tissue (300 mg) was homogenized in lysis buffer containing 10 mM Tris pH 7.4, 3 mM MgCl_2_ and protease inhibitors (Roche Diagnostics, Basel, Switzerland). The homogenates were sonicated and centrifuged (1,000 × *g* for 5 min at 4°C) to separate the adipocytes, followed by a second spin at 12,000 × *g* for 15 min. Total protein concentration in the supernatants was determined with bicinchoninic acid protein assay kit (Pierce, Thermo Fisher Scientific). 50 µg of protein from each sample was loaded into a 96-well plate in duplicate and assayed according to the manufacturer’s instructions using a Bio-Plex 200 system (Bio-Rad Laboratories, Hercules, CA, USA).

### Analysis and Statistical Methods

For flow cytometry, leukocytes were isolated from approximately 300 mg of adipose tissue from each animal. Absolute counts were calculated by normalizing cell events to bead counts and adipose tissue weights. Statistical analysis was performed using two-way ANOVA with sex and age/treatment as the independent factors. *Post hoc* analysis was performed with Sidak’s multiple comparisons test. Data from experiments using middle-aged animals only were analyzed with unpaired *t*-test or unpaired *t*-test with Welch’s correction when variances were unequal. Statistical significance was considered at *p* < 0.05. All statistical analyses were performed with GraphPad Prism 7.

## Results

### Age-Associated Increase in Adipose Tissue Mass and Shifted Lipid Profiles in Middle-Aged Females

**Figure 1:**
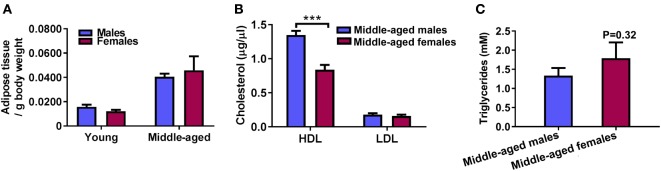
Age-induced increase in adipose tissue mass and negatively shifted lipid profile in middle-aged females. **(A)** Perigonadal (epididymal in males and parametrial in females) adipose tissue mass normalized to body weight in young (3–4 months) and middle-aged (15–16 months) mice. Two-way ANOVA effect of age: *p* < 0.001, *n* = 9. **(B)** Plasma high-density lipoprotein (HDL) cholesterol and low-density lipoprotein cholesterol (LDL) in middle-aged mice. ***p < 0.001 unpaired *t*-test, *n* = 10. **(C)** Plasma triglycerides in middle-aged male and female mice. *p* = 0.32 unpaired *t*-test, *n* = 9–10.

Middle-aged mice at 15–16 months of age and young mice of 3–4 months of age were used in this study. Initially, we evaluated the translational relevance of our middle-aged male and female mice to study obesity-induced adipose tissue inflammation. A significant age-associated increase in body weight (not shown) and adipose tissue mass was observed in middle-aged males and females compared to young mice of the same sex (Figure 1A, two-way ANOVA, effect of age: *p* < 0.001). No significant male vs female difference in body weight was seen in middle-aged mice (males: 40 ± 1 vs females: 35 ± 3 g, *n* = 10) while young males weighed more than young females (29 ± 1 vs 21 ± 1 g, *p* < 0.05, *n* = 9). Plasma TGs and HDL/LDL cholesterol levels were measured in middle-aged mice to determine the lipid profiles in these animals. Middle-aged females had lower levels of plasma HDL compared to males, a characteristic of dyslipidemia in humans (23), with no effect of sex seen on LDL cholesterol or TG levels (Figures 1B,C, *n* = 10). Lastly, we confirmed that middle-aged female mice used in our model were reproductively senescent. Similar to other studies, we observed an increase in uteri size in middle-aged females due to fibrosis and collagen deposition (Figure S1 in Supplementary Material) (24). We monitored the estrus cycle in young and middle-aged female mice. Vaginal smears were used to verify that young female mice were cycling while middle-aged females were acyclic and showed leukocyte-dominant diestrus-like smears (representative images in Figure S2 in Supplementary Material).

### Female Sex Augments the Age-Associated Increase in Adipose Tissue CD8^+^ T Cells

**Figure 2:**
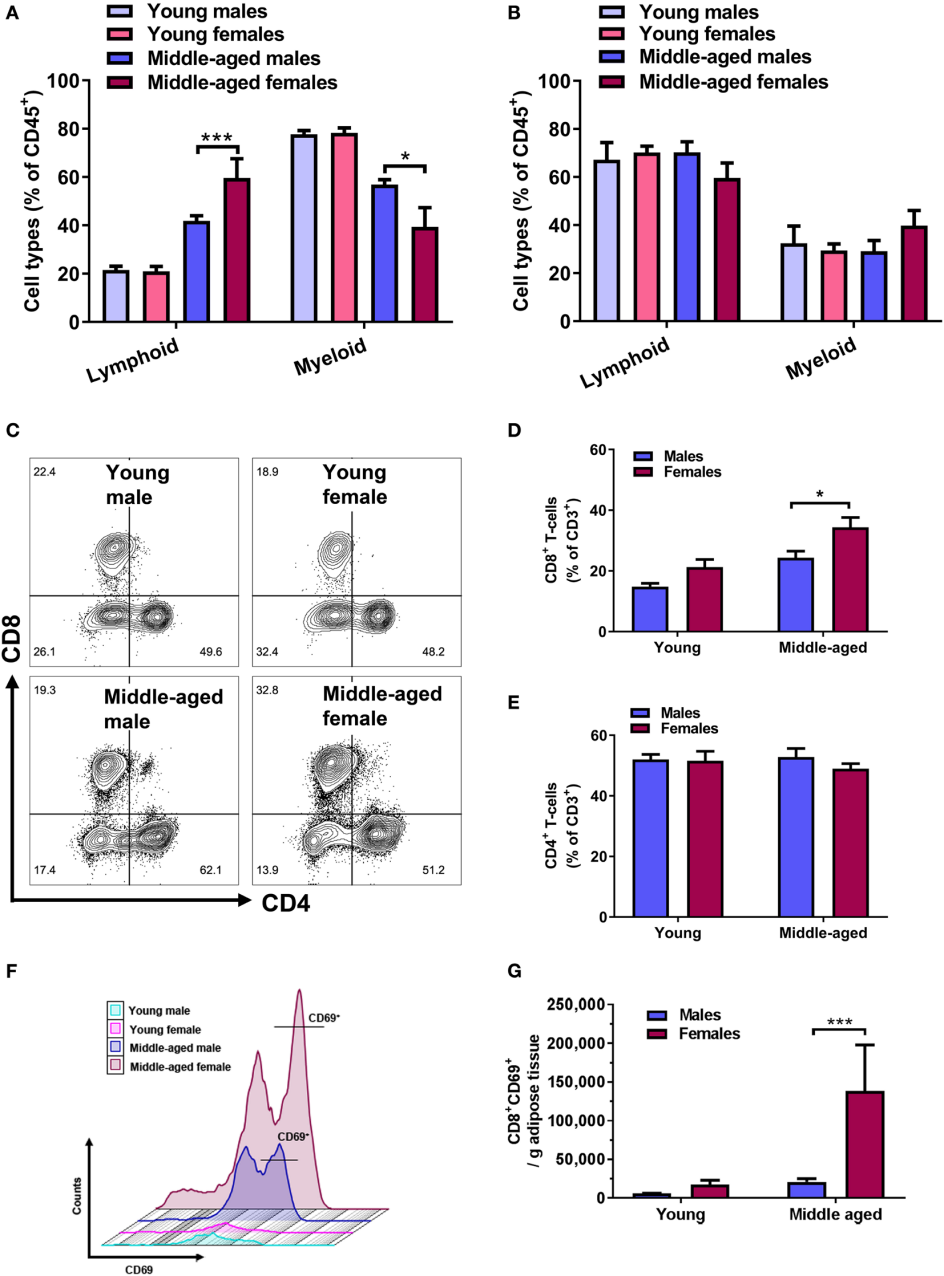
Age-associated increases in adipose lymphoid cells and CD8^+^ T cells are pronounced in females. Flow cytometry quantification of lymphoid (CD45^+^CD11b^−^) and myeloid cell (CD45^+^CD11b^+^) percentages of **(A)** adipose tissue and **(B)** blood-derived immune cells. Two-way ANOVA lymphoid cells: effect of age *p* < 0.001, effect of sex *p* < 0.01; myeloid cells: effect of age *p* < 0.001, *p < 0.05, ***p < 0.001 Sidak’s multiple comparison’s test, *n* = 5. **(C)** Representative contour plots of CD4^+^ and CD8^+^ T-cell populations, with outliers displayed, in adipose tissue of young and middle-aged male and female mice. **(D)** Quantification of CD8^+^ T-cell percentages (CD3^+^CD8^+^) and **(E)** CD4^+^ T cells (CD3^+^CD4^+^) in adipose tissue of young and middle-aged mice using flow cytometry. Two-way ANOVA effect of age: *p* < 0.001, sex: *p* < 0.01. *p < 0.05 Sidak’s multiple comparison’s test, *n* = 8–9. **(F)** Representative histograms of CD8^+^CD69^+^ T cells in adipose tissue from young and middle-aged male and female mice. **(G)** Absolute number of activated CD8^+^ T cells (CD8^+^CD69^+^) normalized to bead counts and adipose tissue weights. Two-way ANOVA effect of age: *p* < 0.05, sex: *p* < 0.05, ***p < 0.001 Sidak’s multiple comparison’s test, *n* = 5.

Analysis of adipose tissue-derived immune cells using flow cytometry showed that there was no difference in the number of lymphoid and myeloid cells between young male and female mice (Figure 2A, *n* = 5). However, we found a significant increase in the lymphoid cells (*p* < 0.001), and a decrease in myeloid cells with age (*p* < 0.001), which was significantly more pronounced in females (Figure 2A). This change in the lymphoid-to-myeloid proportion in middle-aged females was mainly driven by a significant increase in lymphocyte cell counts (Figure S3 in Supplementary Material). There was no effect of age and sex on the lymphoid-to-myeloid cell ratio in the blood circulation, with lymphoid cells representing the dominant subset in all experimental groups (Figure 2B, *n* = 5).

Next, we tested whether the age-related increase in adipose tissue lymphoid cell populations was associated with changes in any specific T lymphocyte subpopulation. Flow cytometry was used to identify adipose tissue CD4^+^ and CD8^+^ T-cell populations in young and middle-aged mice of both sexes. Representative contour plots of adipose CD4^+^ and CD8^+^ T-cell populations, with outliers, from young and middle-aged mice are shown in Figure 2C. Middle-aged mice overall, and females especially, showed a greater age-associated increase in CD8^+^ T cells compared to young mice (% increase compared to young, females: 86 ± 5, males: 66 ± 8, *p* ≤ 0.05). The proportion of adipose CD8^+^ T cells was larger in middle-aged females than in males (Figure 2D, *p* < 0.05). No significant age or sex differences in CD4^+^ T cells were observed (Figure 2E). When we measured CD8^+^ T-cell activation, we found that middle-aged females had significantly more CD8^+^ T cells that expressed CD69, a marker indicative of early T-cell activation (Figures 2F,G, *p* < 0.001). Analysis of CD4^+^ and CD8^+^ T-cell populations in the spleen as a comparison showed a trend toward lower levels of CD8^+^ T cells in young females that reached significance in the middle age (Figure S4A in Supplementary Material). In line with this finding, the proportion of CD4^+^ T cells was significantly higher in middle-aged females (Figure S4B in Supplementary Material).

### Sex Differences in CD8^+^ T-Cell-Associated Cytokines in Adipose Tissue

**Figure 3:**
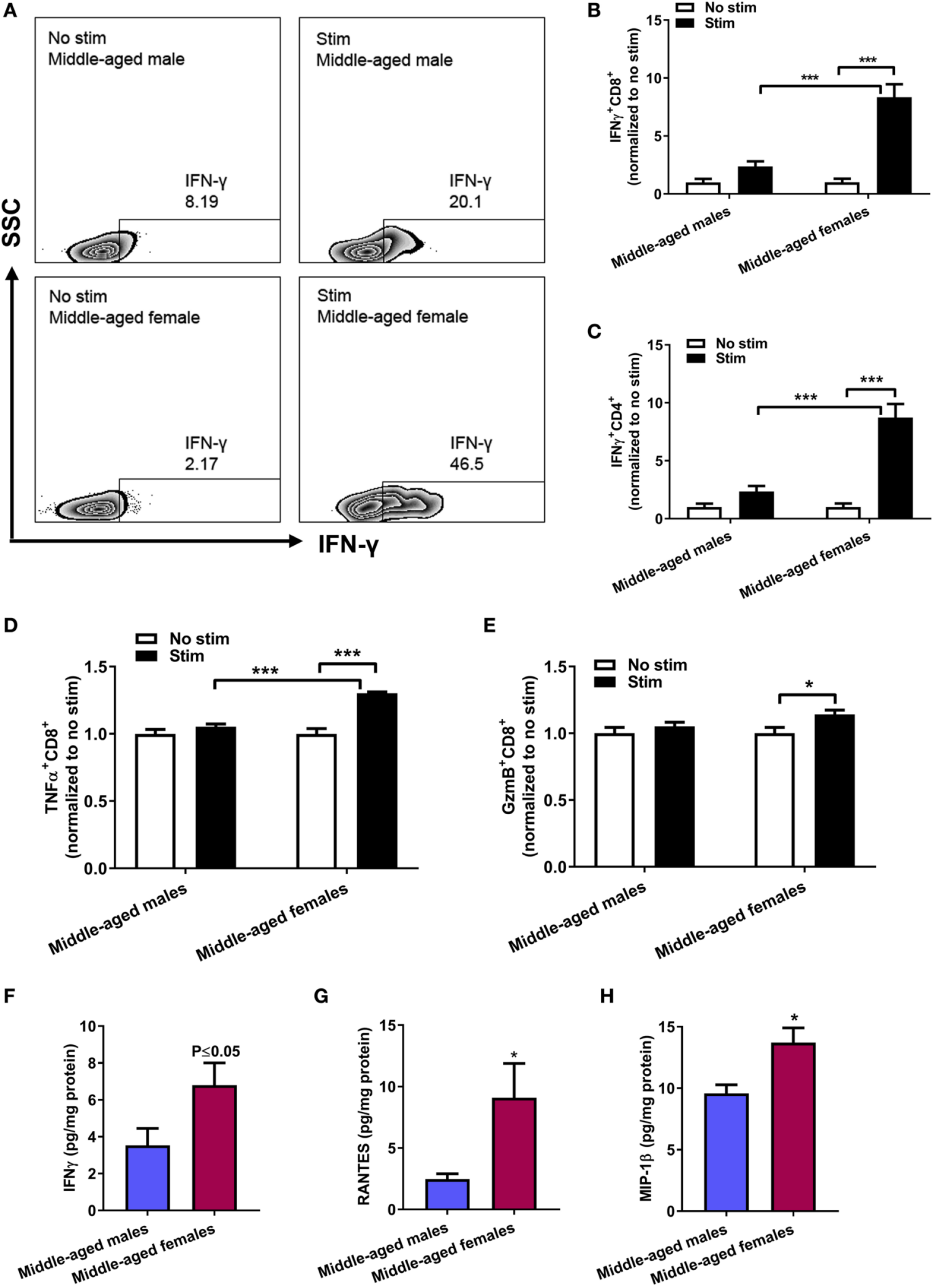
Sex differences in adipose CD8^+^ T-cell cytokine production *ex vivo* and total adipose cytokine levels. **(A)** Representative contour plots of IFN-γ^+^CD8^+^ T cells after *ex vivo* stimulation with phorbol 12-myristate 13-acetate and ionomycin for 4 h at 37°C 5% CO_2_. Quantification of **(B)** IFN-γ^+^CD8^+^ T cells, **(C)** IFN-γ^+^CD4^+^ T cells, **(D)** TNF-α^+^CD8^+^ T cells, and **(E)** Granzyme B (GzmB)^+^CD8^+^ T cells normalized to no stimulation conditions within each sex. Two-way ANOVA, *p < 0.05, ***p < 0.001 Sidak’s multiple comparison’s test, *n* = 5. Total adipose levels of **(F)** IFN-γ, **(G)** RANTES, and **(H)** MIP-1β in middle-aged mice by multiplex cytokine measurement. *p* ≤ 0.05 unpaired *t*-test, *p < 0.05 unpaired *t*-test with Welch’s correction, *n* = 8–10.

Having demonstrated that middle-aged females exhibited a pronounced age-induced increase in activated CD8^+^ T cells, we characterized their phenotype. *Ex vivo* stimulation assay using PMA and ionomycin showed a strong induced production of IFN-γ in CD8^+^ T cells from middle-aged females that was significantly larger compared to middle-aged males (Figures 3A,B, two-way ANOVA, stim middle-aged females vs stim middle-aged males: *p* < 0.001, *n* = 5). Similar results were obtained for IFN-γ^+^ CD4^+^ T cell after stimulation (Figure 3C). We further measured the induced response of CD8^+^ T cells to produce TNF-α and granzyme B (GzmB), effector mechanisms of CD8^+^ T cells in mediating target cell lysis and apoptosis. Stimulation with PMA/ionomycin induced a significant increase in TNF-α^+^ CD8^+^ T cells in middle-aged females (no stim vs stim, *p* < 0.001, *n* = 5) that was significantly higher than in middle-aged males after stimulation (*p* < 0.001, Figure 3D). Likewise, the stimulation only led to increased GzmB production in middle-aged females and not in middle-aged males (*p* < 0.05) (Figure 3E).

Supporting the IFN-γ finding in our *ex vivo* studies, analysis of cytokine levels in adipose tissue homogenate showed higher levels of IFN-γ (Figure 3F, *p* ≤ 0.05, *n* = 8–10) in female adipose tissue. Lastly, we measured the levels of RANTES (CCL5), a known CD8^+^ T-cell chemokine and ligand for the CCR5 receptor, and macrophage inflammatory protein 1β (MIP-1β, CCL4), another chemokine with specificity for the CCR5 receptor. Adipose tissue homogenates of middle-aged females showed higher levels of both RANTES and MIP-1β compared to age-matched males (Figures 3G,H, *p* < 0.05).

### Higher Levels of Adipose Tregs in Males Compared to Age-Matched Females

**Figure 4:**
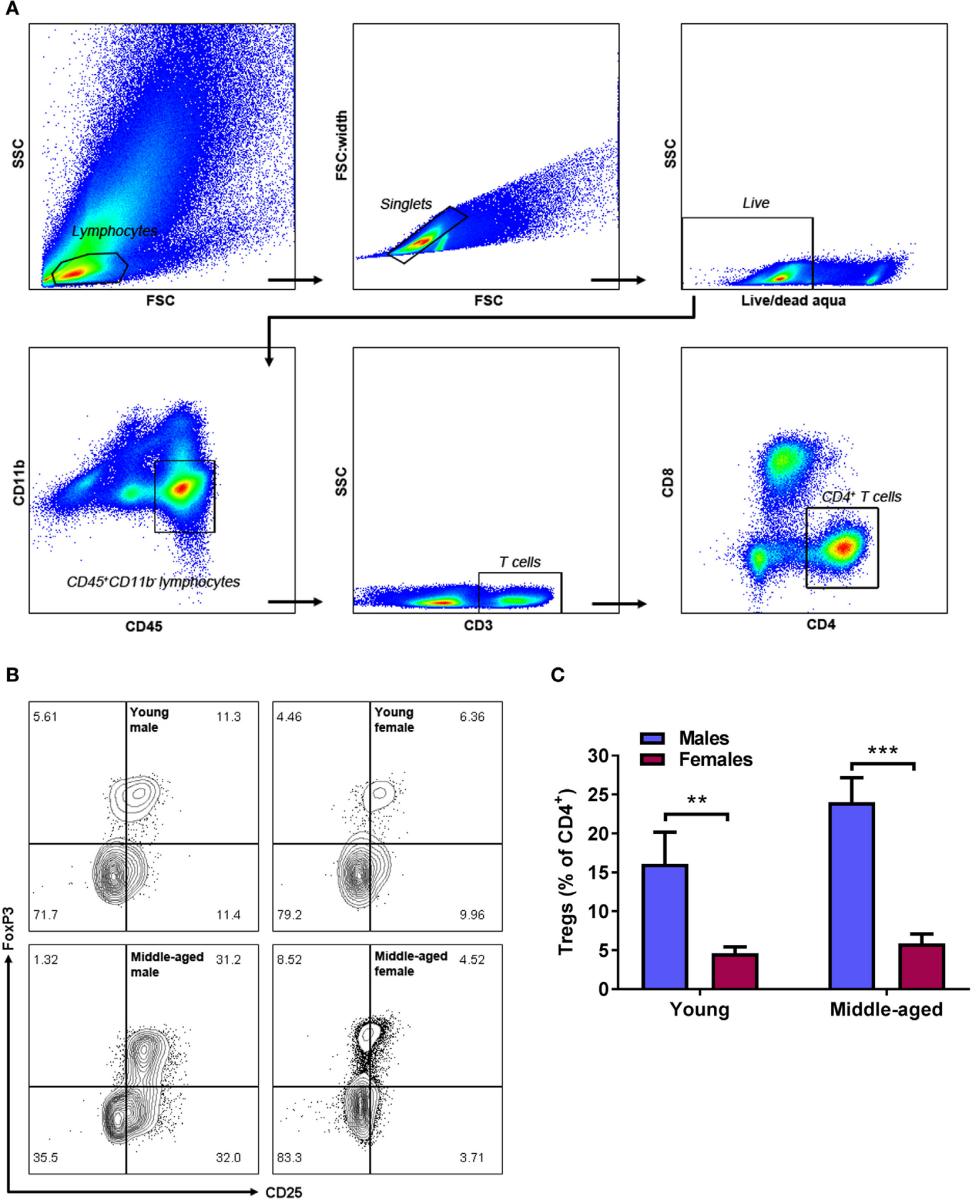
Males have higher levels of adipose regulatory T cells (Tregs) compared to age-matched females. Gating strategy **(A)**, representative contour plots **(B)**, and quantification **(C)** of CD4^+^CD25^+^FoxP3^+^ Tregs in adipose tissue from young and middle-aged male and female mice. Two-way ANOVA effect of sex: *p* < 0.001, **p < 0.01, ***p < 0.001 Sidak’s multiple comparison’s test, *n* = 9.

Regulatory T cells is a sub-population of CD4^+^ T cells that express the T-cell activation marker CD25 and nuclear FoxP3 (gating strategy and representative contour plots in Figures 4A,B). In contrast to the age-associated increase in adipose CD8^+^ T cells in middle-aged females, no change in anti-inflammatory Tregs with age was observed (Figure 4C, *n* = 9). Independent of their age, females had significantly less adipose Tregs compared to age-matched males (two-way ANOVA, effect of sex: *p* < 0.001, Figure 4C). Thus, in the context of increasing pro-inflammatory CD8^+^ T cells, this lack of Treg increase in middle-aged females results in a shift in balance to the pro-inflammatory T-cell phenotype.

## Discussion

In the present study, we demonstrate that middle-aged mice of both sexes have significantly higher levels of adipose tissue CD8^+^ T cells than young mice. Interestingly, middle-aged female mice have a greater age-related increase in adipose CD8^+^ T cells compared to middle-aged males. No effects of sex or age were seen in adipose CD4^+^ T-cell levels, suggesting that age-related adipose tissue T-cell infiltration may be subset specific.

**Figure 5:**
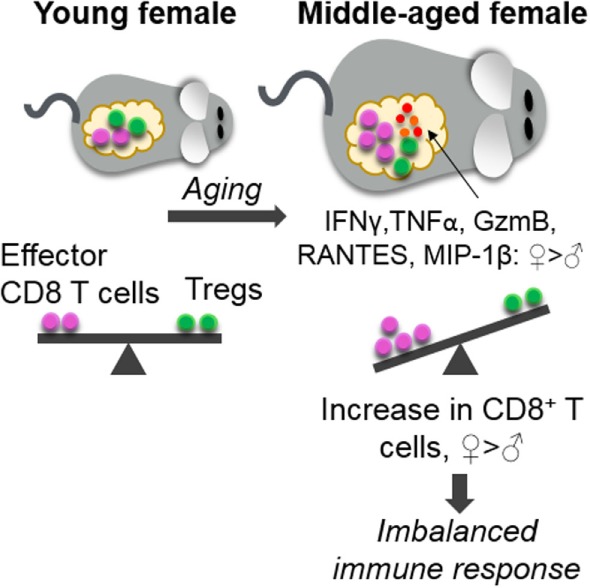
Age-associated increase in adipose CD8^+^ T cells and imbalanced immune response in middle-aged females. Middle-aged females had a greater increase in adipose effector CD8^+^ T cells than males, without a compensatory increase in anti-inflammatory regulatory T cells (Tregs). Higher levels of the CD8^+^ T-cell chemokine RANTES, MIP-1β, and the pro-inflammatory cytokines IFN-γ, TNF-α, and granzyme B (GzmB) were also observed in middle-aged females. We hypothesize that this imbalance may promote a pro-inflammatory milieu and contribute to increased cardiovascular disease burden in aging females.

Importantly, middle-aged females had significantly higher numbers of activated (CD69^+^) CD8^+^ T cells in adipose tissue when compared to middle-aged male mice and young mice of both sexes, suggesting that these cells are in a basally activated state. Following *ex vivo* stimulation, CD8^+^ T cells from the adipose tissue of middle-aged female mice produced significantly higher levels of intracellular IFN-γ, TNF-α, and GzmB than their male counterparts confirming that these T cells have a stronger pro-inflammatory phenotype. Multiplex cytokine measurements further showed higher levels of IFN-γ, RANTES, and MIP-1β in adipose tissue harvested from females. In parallel, we demonstrate that middle-aged females have lower levels of Tregs, a pro-homeostatic immune cell that normally decreases in number in mice and humans in obesity (17, 18). The balance shift of high levels of inflammatory CD8^+^ T cells and low levels of anti-inflammatory Tregs may promote an overall pro-inflammatory milieu in aging females (Figure 5) that could have adverse adipose consequences.

The rising incidence of obesity poses a major health risk worldwide and is especially a concern in the aging population (5). Almost a decade ago, a series of experimental studies suggested the importance of CD8^+^ T cells in obesity and adipose tissue inflammation, especially during early stages of obesity development (15–17, 25). CD8^+^ T-cell infiltration into adipose tissue preceded the infiltration of macrophages and was proposed to be responsible for the initiation and propagation of adipose tissue inflammation (16). It was also shown that CD8^+^ T cells in adipose tissue exhibit an activated phenotype, characterized by increased proliferation and augmented expression of IFN-γ (26). While most of the studies pertaining to adipose inflammation were performed in young male mice and utilized either genetic- or diet-induced models of obesity, studies comparing sex or addressing age as a factor are missing in the literature. Furthermore, it has been shown that diet-induced obesity induces different types of adipose tissue inflammation than what is observed with natural age-induced obesity (27). In addition, by using diet- or genetic-induced obesity, the body and vasculature of the animal is still young, rather than middle aged. These points are critically significant because obesity may be more common in aging women and may confer greater risk for secondary health issues such as ischemic stroke (1, 4, 5, 8, 9).

Consistent with previous studies (15, 16, 26), we confirm that increased adipose tissue mass (here, due to aging) is associated with increased number of adipose tissue lymphocytes, particularly CD8^+^ T cells. To our knowledge, our present studies provide the first evidence that female sex augments the age-induced increase in adipose CD8^+^ T-cell number and activation compared to middle-aged males. Female sex was also found to augment the pro-inflammatory T-cell phenotype in middle-aged animals, as activated CD8^+^ T cells harvested from the adipose tissue of female animals produced higher levels of IFN-γ, TNF-α, and GzmB when stimulated with PMA and ionomycin *ex vivo*. These cytokines and lytic enzymes are an essential part of cytotoxic CD8^+^ T-cell-mediated mechanisms of action. Upon recognition of antigens on the surface of target cells, the main act of cytotoxic T cells is to release specialized lytic granules containing perforin and granzymes to kill the target cells. CD8^+^ T cells also act by releasing cytokines IFN-γ, TNF-α, and TNF-β to recruit and activate macrophages (28). Hence, our *ex vivo* stimulation data show that the main effector mechanisms of CD8^+^ T cells were potentiated in middle-aged females. As further *in vivo* support for this finding, cytokine measurement in adipose tissue homogenates showed higher levels of IFN-γ in middle-aged females compared to males.

Regulatory T cells have been shown to play an important role in the downregulation of inflammation in obesity. Obese human subjects have lower levels of circulating Tregs and decreased mRNA expression of the Treg-specific FoxP3 transcript in omental adipose tissue compared to normal weight controls (17, 18). The phenomenon of decreased Treg numbers in adipose tissue has been replicated in mice using both genetic- and diet-induced obesity models (17). These earlier studies have also demonstrated that acute depletion of Tregs results in increased transcription of inflammatory genes in adipose tissue, suggesting that Tregs play an important role in suppressing obesity-related inflammation. Our data show that, independent of age, females had significantly lower number of adipose Tregs than males. The age-induced increase in activated CD8^+^ T cells and production of IFN-γ, TNF-α, and GzmB in females, particularly without a compensatory expansion of Tregs, may create an immune imbalance and foster a pro-inflammatory environment in the adipose tissue of middle-aged females (Figure 5).

The mechanisms underlying the age-induced accumulation of CD8^+^ T cells in adipose tissue, and the greater accumulation and pro-inflammatory activation of CD8^+^ T cells seen in middle-aged females, remain unknown. Our studies demonstrated increased levels of RANTES and MIP-1β in middle-aged females. These chemokines are both ligands that can bind to the CCR5 receptor, an important step in the attraction of T cells to specific tissue, and are shown to be increased in adipose tissue from obese mice and in obese humans (16, 29, 30). MIP-1β has been reported to attract NK cells (31), but our preliminary data show decreased NK cell levels with aging and did not differ between males and females (unpublished data), suggesting that the sex differences in MIP-1β levels most likely are not associated with NK cells. As MIP-1β is produced in large amounts by activated CD8^+^ T cells, our multiplex data are in line with our other results showing a stronger pro-inflammatory state in middle-aged female CD8^+^ T-cell parameters (CD69^+^ activation and the induced production of IFN-γ, TNF-α, and GzmB). RANTES has further been demonstrated to be specifically important for the recruitment and regulation of CD8^+^ T cells (32). Once recruited to adipose tissue, T cells must be activated to sustain a pro-inflammatory phenotype. Our results showing a large number of activated CD8^+^CD69^+^ T cells in adipose tissue of middle-aged females suggest that these cells have a basally active phenotype.

Few studies on obesity-related adipose tissue inflammation have investigated sex differences. In a study by Wu et al., young male and female mice were fed a high-fat diet for 24 weeks (30). The authors showed a greater accumulation of T cells in males, along with an increased expression of RANTES and CCR5, compared to females. Protection from obesity-associated inflammation in young females has been reported by others (33, 34). High-fat diet fed females showed no increase in adipose macrophages, a lack of systemic inflammation, and an expansion of their Treg population, while the opposite effects were seen in young males (34). Estrogen likely contributes to the protection seen in young females, as ovariectomy increases adipose tissue mass and the infiltration of T cells and macrophages (35). Sex chromosomes are also important, as mice with two X chromosomes have increased adiposity and food intake independent of gonadal sex in the four core genotype model (36). Our study in middle-aged animals shows significant sex differences in obesity-related inflammation, which was characterized by an augmented age-induced accumulation of CD8^+^ T cells in the adipose tissue of female animals, higher levels of adipose IFN-γ, TNF-α, GzmB, RANTES, and MIP-1β in female mice, and the absence of an age-related expansion of the adipose tissue Treg population in middle-aged females. The results highlight the importance of using middle-aged and aged animals of both sexes in obesity-related studies.

The shift in adipose T cells seen in middle age could be a potential contributing mechanism to the switch from an “ischemia-protected” phenotype in young female mice to an “ischemia-sensitive” phenotype in middle-aged (3). A connecting link between obesity, adipose tissue inflammation, and inflammation in the brain has been reported in mice and humans (14, 37). Future studies are needed to investigate the adipose-to-brain inflammation axis in females and middle-aged animals.

### Limitations and Future Directions

In the current study, we investigated sex differences in T cells and Tregs from the abdominal adipose tissue depot as several studies show that central obesity is linked to cardiovascular disease such as ischemic stroke, and may contribute to a greater risk in women than in men (1, 8, 9). In addition, abdominal adipose tissue is one of the larger depots which made it possible for us to perform the present flow cytometry experiments and functional assays. However, a recent study suggests that other adipose tissue depots, such as epicardial adipose tissue (EAT), may also present sex differences in obesity and aging (38). It has also been shown that EAT from patients with coronary heart disease had accumulation of CD8^+^ T cells compared to healthy patients (39). In the latter study, no sex-specific analysis was made and sex differences in age-dependent EAT inflammation and immune responses remains to be investigated.

The present study focused on lymphocytes, T cells in particular, in adipose tissue inflammation with aging as these cell populations markedly increase with age. The current study contributes to our knowledge of the established role of CD8^+^ T cells in obesity and augments data from previous studies. However, accumulation of myeloid cells, such as macrophages, in adipose tissue is an important contributor and consequence of obesity and also warrants study. In addition, how this shift in T cells contributes to systemic inflammation and their specific contribution to the response to brain injury remains to be investigated. In particular, we plan to examine the potential causative role of adipose CD8^+^ T cell to Treg balance on stroke outcome in middle-aged mice.

## Conclusion

We propose that increased levels of activated adipose CD8^+^ T cells in combination with low levels of anti-inflammatory Tregs create an imbalance in the pro/anti-inflammatory T-cell milieu and contribute to a “primed” pro-inflammatory environment in middle-aged females. This may render middle-aged females more susceptible to secondary health issues that increase in incidence with aging, such as cardiovascular disease and ischemic stroke.

## Ethics Statement

Animal procedures were performed in accordance with National Institutes of Health Guidelines for the care and use of laboratory animals and approved by the Animal Welfare Committee at the University of Texas Health Science Center at Houston, Texas (AWC-15-0140).

## Author Contributions

HA made substantial contributions to the conception, design, and drafting of the work and performed all experiments. JA, SM, LM, and MR-O provided substantial contribution to the design of the work as well as revising for important intellectual content. MR-O, MS, AM, JB-A, and AC contributed to the flow cytometry experiments and revised the manuscript. All authors have read and approved the final version to be published.

## Conflict of Interest Statement

The authors declare that the research was conducted in the absence of any commercial or financial relationships that could be construed as a potential conflict of interest.
