# Supplementary material for: Sex Differences in Adipose Tissue CD8+ T Cells and Regulatory T Cells in Middle-Aged Mice
Source: Front Immunol. 2018 Apr 4;9:659. doi: 10.3389/fimmu.2018.00659 (PMC5893719; doi:10.3389/fimmu.2018.00659)
Supplement: Supplementary file 1 [file Data_Sheet_1.docx]

Supplementary Material

Sex Differences in Adipose Tissue CD8^+^ T Cells and Regulatory T Cells in Middle-Aged Mice

**Hilda Ahnstedt*, Meaghan Roy-O’Reilly, Monica S Spychala, Alexis S Mobley, Javiera Bravo-Alegria, Anjali Chauhan, Jaroslaw Aronowski, Sean P Marrelli and Louise D McCullough**

*** Correspondence:** Corresponding Author: [Hilda.W.Ahnstedt@uth.tmc.edu](mailto:hilda.W.ahnstedt@uth.tmc.edu)

## Supplementary Figures

**
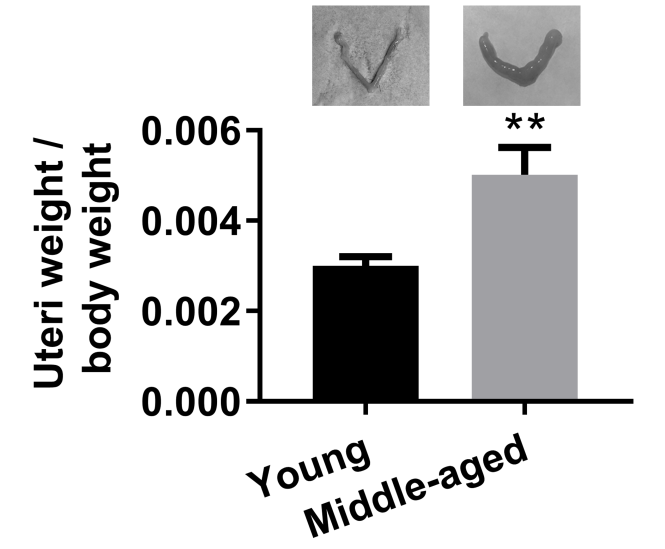
**

**Supplementary Figure 1.** Uteri weight in young and middle-aged female mice. Wet-weights of uteri normalized to body weight. **p<0.01, unpaired t-test, n=6-10.


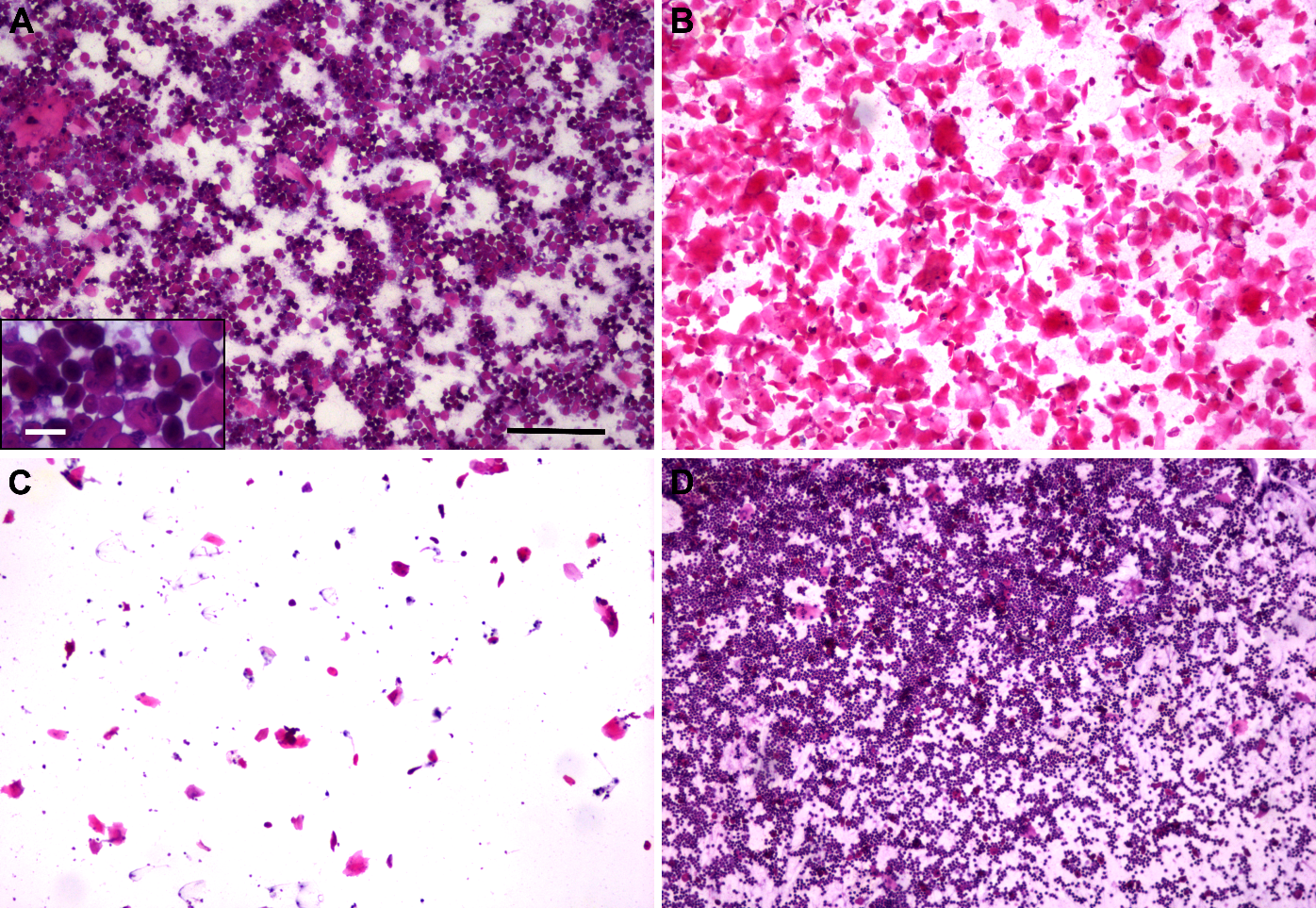


**Supplementary Figure 2.** Representative images of hematoxylin and eosin-stained vaginal smears from mice at **(A)** pro-estrus, predominantly consisting of nucleated epithelial cells, **(B)** estrus, with cornified cells lacking a nucleus, **(C)** metestrus, consisting of nucleated epithelial cells, cornified cells and leukocytes, and **(D)** diestrus, mainly consisting of leukocytes. Young female mice (3-4 months) showed regular cyclicity with one day of pro-estrus, one to two days of estrus, one day of metestrus followed by diestrus before re-starting the cycle. 15-16 month old mice were acyclic and showed leukocyte-dominant diestrus-like smears. Scale bar: 50 µm, insert scale bar: 25 µm.


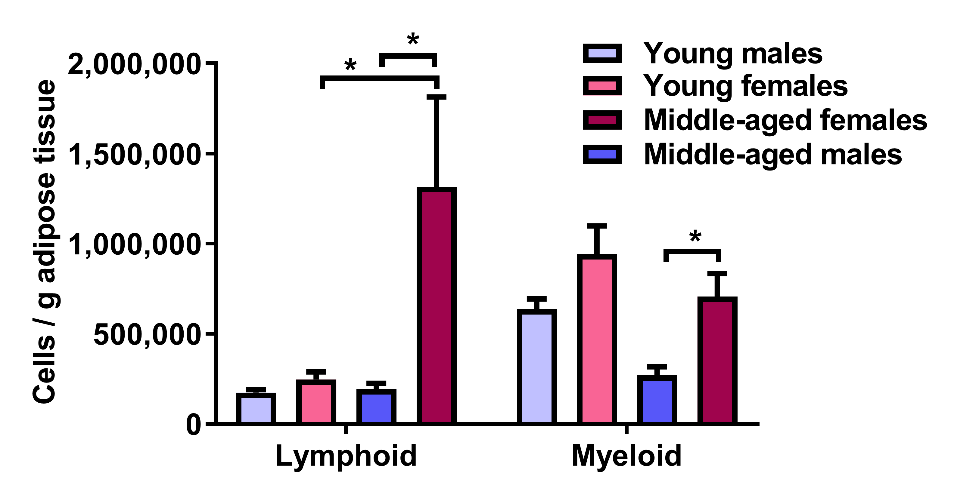


**Supplementary Figure 3.** Absolute lymphoid (CD45^+^CD11b^-^) and myeloid (CD45^+^CD11b^+^) cell counts in young and middle-aged male and female mice. Two-way ANOVA lymphoid/myeloid cells: effect of sex p<0.05, effect of age: p<0.05, *p<0.05 Sidak’s multiple comparison’s test. n=5


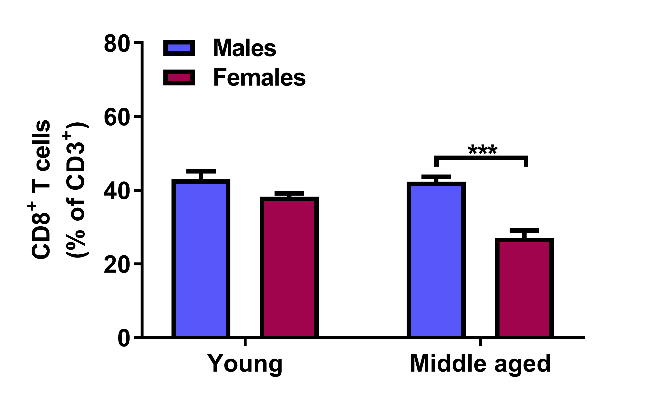

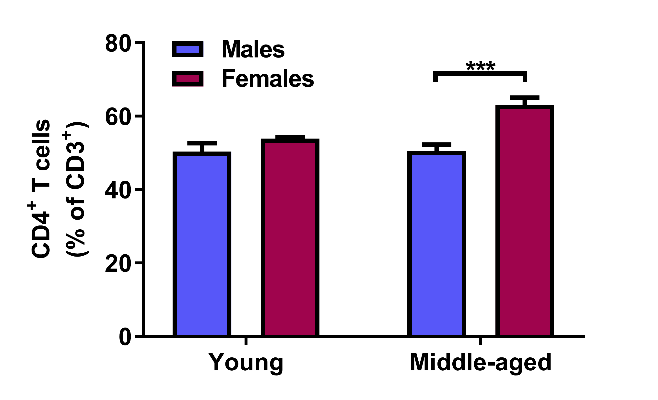


**Supplementary Figure 4.** Sex differences in splenic CD8^+^ and CD4^+^ T cell proportions from middle-aged mice. **(A)** Middle-aged females had lower levels of CD8^+^ T cells in the spleen. Two-way ANOVA, effect of sex: p<0.001, effect of age: p<0.01. ***p<0.001 Sidak’s multiple comparison’s test. n=8-9. **(B)** Middle-aged females showed significantly higher levels of CD4^+^ T cells compared to males. Two-way ANOVA, effect of sex: p<0.001, effect of age: p<0.05. ***p<0.001 Sidak’s multiple comparison’s test. n=8-9.
